# Within- and between-therapist agreement on personalized parameters for robot-assisted gait therapy: the challenge of adjusting robotic assistance

**DOI:** 10.1186/s12984-023-01176-x

**Published:** 2023-06-20

**Authors:** Florian van Dellen, T. Aurich-Schuler, Rob Labruyère

**Affiliations:** 1 Sensory-Motor Systems Lab, Department of Health Sciences and Technology, ETH Zurich, Tannenstrasse 1, 8092 Zurich, Switzerland; 2 Swiss Children’s Rehab, University Children’s Hospital Zurich, Mühlebergstrasse 104, 8910 Affoltern Am Albis, Switzerland; 3 Children’s Research Center, University Children’s Hospital Zurich, Steinwiesstrasse 75, CH-8032 Zurich, Switzerland

**Keywords:** Bodyweight support, Gait speed, Guidance force, Path control, Exoskeleton, Therapist decision making

## Abstract

**Background:**

Stationary robotic gait trainers usually allow for adjustment of training parameters, including gait speed, body weight support and robotic assistance, to personalize therapy. Consequently, therapists personalize parameter settings to pursue a relevant therapy goal for each patient. Previous work has shown that the choice of parameters influences the behavior of patients. At the same time, randomized clinical trials usually do not report the applied settings and do not consider them in the interpretation of their results. The choice of adequate parameter settings therefore remains one of the major challenges that therapists face in everyday clinical practice. For therapy to be most effective, personalization should ideally result in repeatable parameter settings for repeatable therapy situations, irrespective of the therapist who adjusts the parameters. This has not yet been investigated. Therefore, the aim of the present study was to investigate the agreement of parameter settings from session to session within a therapist and between two different therapists in children and adolescents undergoing robot-assisted gait training.

**Methods and results:**

Fourteen patients walked in the robotic gait trainer Lokomat on 2 days. Two therapists from a pool of 5 therapists independently personalized gait speed, bodyweight support and robotic assistance for a moderately and a vigorously intensive therapy task. There was a very high agreement within and between therapists for the parameters gait speed and bodyweight support, but a substantially lower agreement for robotic assistance.

**Conclusion:**

These findings imply that therapists perform consistently at setting parameters that have a very clear and visible clinical effect (e.g. walking speed and bodyweight support). However, they have more difficulties with robotic assistance, which has a more ambiguous effect because patients may respond differently to changes. Future work should therefore focus on better understanding patient reactions to changes in robotic assistance and especially on how instructions can be employed to steer these reactions. To improve the agreement, we propose that therapists link their choice of robotic assistance to the individual therapy goals of the patients and closely guide the patients during walking with instructions.

**Supplementary Information:**

The online version contains supplementary material available at 10.1186/s12984-023-01176-x.

## Background

The improvement of walking functions is a major rehabilitation goal in neuropaediatric patients [1]. Rehabilitation research has established that goal-oriented therapy [2], lots of practice [3], and the patient’s active participation [4] are important contributors to a successful rehabilitation process. Therapists commonly use these motor learning principles to adapt therapy content to the needs of individual patients. However, scientific literature provides little evidence on the personalization of therapies. This can be partially attributed to the fact that describing therapy content in a rehabilitation context is challenging [5]. Whereas in the pharmaceutical context, therapy content can be clearly described by formulation, dose, and dosage, active rehabilitation interventions are not as clearly defined and can include a range of different activities/exercises/movements chosen by the therapist for specific patients. As a consequence, specific information about the therapy content is missing in many studies evaluating the effectiveness of physiotherapeutic interventions [6]. This applies also to modern robotic gait therapies [7, 8]. In the case of conventional therapies, reasons for the lack of information include missing tools which can quantify and describe the therapist-patient interactions. However, in modern robotic systems, control parameters set by the therapist and sensors measuring the patient's behavior allow for the quantification, storage, and study of information on therapist-patient interactions mediated by the device. Nevertheless, this information has been rarely used to better understand the therapist’s decision making process.

One example of such a platform is the widely used exoskeleton Lokomat (Hocoma AG, Volketswil, Switzerland [9]. The Lokomat provides the therapist with several parameters that can be tuned to challenge patients individually [10, 11]. The most commonly used parameters are the regulation of gait speed, the amount of unloading via a harness called the bodyweight support, and a scaling factor for the force field, that keeps the hip and knee joints on the desired angular trajectory, called the Guidance Force [12]. In addition, the robotic assistance can also be tuned with a newer control mode named Path Control to allow for temporal variability [11]. It is known from research focusing on the device-patient interaction that all these parameter settings can influence patient behavior [11, 13, 14]. The desired physiologic reaction, which depends on the therapy goal, may vary between patients due to factors such as the day's condition, the underlying disease, motivation, or the relationship with the therapist. Therefore, the optimization of parameter settings cannot be performed with a single objective function for all patients, but different objectives must be carefully selected and weighted. Consequently, finding optimal parameter settings remains an important task in the responsibility of the therapist. That therapists perform well at this task is a key assumption of many clinical studies [15–19]. A consistent strategy is critical to ensure the validity and repeatability of this type of therapy in clinical practice and a prerequisite for the generalizability of clinical studies results. To the best of our knowledge, no study has investigated the agreement of the choice of parameter settings between and within therapists.

Therefore, the aim of the present study was to investigate the agreement on Lokomat parameter settings from session to session within a therapist and between two different therapists in a pediatric population. To this end, we developed a cross-sectional study protocol asking therapists to individualize therapy parameters for two therapy situations mimicking a real world therapy scenario. Our hypothesis was that the chosen parameter settings agree well within a single therapist and between therapists who received a similar training, with a higher agreement within therapists than between therapists.

## Methods

Ethical approval for the study was obtained from the Cantonal Ethics Committee Zurich (BASEC Nr. 21-D0044), and the study procedures were in accordance with the Declaration of Helsinki.

### Participants

Children and adolescents with a neurologic gait disorder were recruited by convenience sampling between November 2021 and March 2022. Patients between 5 and 18 years old were included. Exclusion criteria were the presence of any factor that prevented the usage of the Lokomat as specified in the device’s handbook [20]. Patients were also excluded if they were unable to follow the study instructions or to communicate pain and discomfort. Written informed consent was provided by the legal guardian of each participant and by the participants themselves if they were 12 years or older.

### Study procedures

**Fig. 1:**
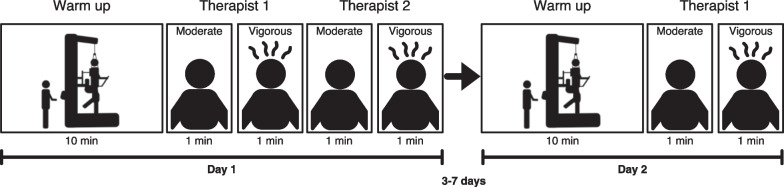
Study design: participants walked for 10 min. in the Lokomat to accommodate themselves. Then, therapists were asked to personalize the device parameters to two target intensities by choosing an appropriate combination of gait speed, bodyweight support, and robotic assistance. After the therapists confirmed their choice, participants walked for 1 min. with the selected parameters. The parameters selected by Therapist 1 and Therapist 2 on Day 1 were used to calculate between-therapist agreement. 3–7 days after the first visit Therapist 1 repeated the task to evaluate within-therapist agreement

The study participants were asked to walk with the LokomatPro Version 6 (Hocoma, Volketswil, Switzerland) on two days. Each of the visits contained about 20 min. of actual walking time. During the first 10 min., patients could accommodate themselves to the device. Two different comparisons were performed: (1) Between-therapist agreement was assessed on the first day by asking two therapists to independently set Lokomat parameters (see Fig. 1). Within-therapist agreement was assessed by asking the first therapist to re-set the parameters at a second visit 3–7 days apart (see Fig. 1). During the parameter setting, therapists were blinded to previously selected parameters by themselves and their peer. The duration of 3–7 days was chosen as a trade-off between a possible memorization of the parameter settings by the therapist and training effects of the patient that could influence the results.

Pairs from the five therapists from the robotics team at the Swiss Children’s Rehab were involved in the study procedures. Thus, 10 different combinations of pairings were possible. All therapists received a similar training and were experienced in Lokomat therapy. The education concept at the Swiss Children’s Rehab includes 5 sessions of one on one training on the device with a test person without impairments, followed by a minimum of 10 sessions of supervised therapies. The first three months include at least 4 therapies per day and a regular exchange with supervisors. Supervisors are therapists with several years of experience and an additional education in gait analysis. The therapy team meets every 2–4 weeks to discuss new literature and therapy approaches.

For the present study, comparable test conditions were created, that mimic real-world therapy situations, by giving therapists a defined therapy goal (“reproduce a physiological gait pattern”) and two different target intensities. They were instructed to set the Lokomat parameters as follows: The target intensity of the first condition was “Moderate”, such that the patient would be able to walk for 30 min. while maintaining a physiological gait pattern. The target intensity of the second condition was “Vigorous”, such that the patient would be able to maintain a physiologic gait pattern for a maximum of 5 min. These two conditions allow to investigate both, baseline parameter settings as well as parameter settings with which therapists try to repeatedly challenge patients for short periods.

The therapists could modify three parameters in this study, namely gait speed, bodyweight support, and the robotic assistance (Table 1). Robotic assistance in the Lokomat consists of two superposing components, namely Guidance Force and Path Control, whereby Path Control is only enabled below 50% Guidance Force. To obtain a single value for robotic assistance, therapists were instructed to work with Guidance Force until 40% and then switch to Path Control to further decrease robotic assistance. To quantify total robotic assistance in this study, Guidance Force and Path Control were added to a single compound parameter of robotic assistance ranging from 200 to 40%. Values between 200 and 140% were achieved by adjusting Guidance Force and values from than 140 to 40% by adjusting Path Control. As an example, if a therapist chooses a Guidance Force level of 40% and a Path Control level of 70%, this would result in a robotic assistance of 110% (40% + 70%). Once the therapists confirmed their choice, the patients walked for one minute with the selected parameters. This phase ensured that the parameters were feasible, especially in the vigorous condition, and that participants could actually walk without stumbling or safety stops. If necessary, therapists were allowed to instruct the patients with a set of standardized sentences (Additional file 1). The final parameters were noted down for analysis.

**Table 1:** Available parameter choices

		Minimum		Maximum	Minimal increment
Gait speed		0.5 km/h		3.6 km/h	0.1 km/h
Bodyweight support		0%		100%	1%
Robotic assistance	Guidance force		40%	100%	5%
	Path control	0%	100%		10%

### Data analysis

All statistical analyses were performed in RStudio (RStudio Team, 2015. RStudio: Integrated Development for R. RStudio, Inc., Boston, MA, USA). The within-therapist and between-therapist agreement was evaluated as follows: For each parameter and condition, thresholds that cover 50%, 75% and 100% of the absolute differences were calculated. In addition, Bland–Altman statistics were performed to estimate the agreement by the Limits of Agreement [21]. To evaluate whether the moderate and vigorous condition resulted in different parameter settings, the mean difference over all patients was calculated. The moderate and vigorous conditions were tested for statistical difference with paired t-tests and we applied a Bonferroni correction for multiple comparisons.

## Results

Of the 14 participants, that were included, 11 completed all conditions. For two participants, only one therapist was available due to sickness of therapists or holidays. One patient could not complete the second visit because of wounds at the feet. Therefore, 13 participants were included in the within-therapist analysis and 12 participants were included in the between-therapist analysis. Detailed information about the patients can be found in Additional file 2.

### Within-therapist agreement

**Fig. 2:**
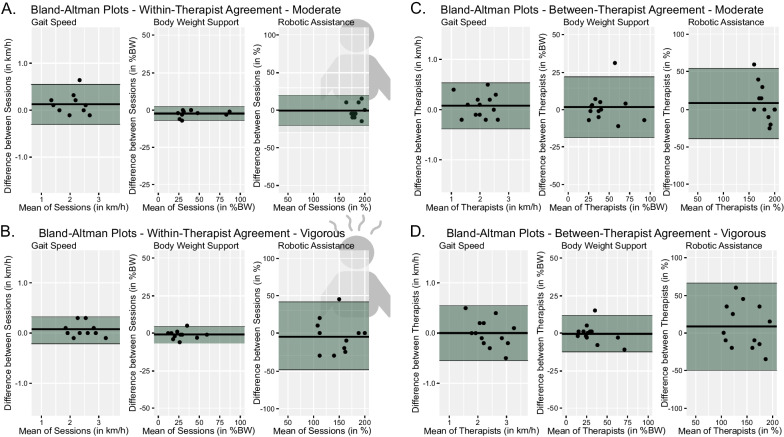
Illustrates the Bland–Altman Plots for the differences of the three parameters per condition. The black lines indicates the mean difference while the green areas mark the Limits of Agreement. **A**, **B** show the within-therapist agreement. **C**, **D** show the between-therapist agreement. The spread of the selected parameters can be seen on the respective x-axis. *BW*  bodyweight

The difference within the same therapist in gait speed was less than 0.2 km/h for the moderate condition and 0.1 km/h for the vigorous condition in at least 75% of the participants (Table 2). Similarly, the difference in bodyweight support was less than 4% of the bodyweight for both conditions (Table 2). The agreement in robotic assistance was lower, especially for the vigorous condition where the difference was bigger than 28% in half of the participants (Table 2). In general, the agreement was high within the same therapist (Fig. 2A, B).

**Table 2:** Statistics of within- and between-therapist agreement

Gait speed (in km/h)	Absolute differences			Bland–Altman statistics	
	< 50%	< 75%	< 100%	Mean	LoA
Within-therapist difference mod	0.1 (2%)	0.2 (5%)	0.6 (14%)	0.10 (2%)	− 0.30–0.50 (− 7–12%)
Within-therapist difference vig	0.1 (2%)	0.1 (2%)	0.3 (12%)	0.04 (1%)	− 0.22–0.31 (− 5–7%)
Between-therapist difference mod	0.2 (5%)	0.2 (5%)	0.5 (12%)	0.07 (2%)	− 0.38–0.54 (− 9–13%)
Between-therapist difference vig	0.2 (5%)	0.3 (7%)	0.5 (12%)	0 (0%)	− 0.54–0.54 (− 13–13%)

Bodyweight support (in %)	< 50%	< 75%	< 100%	Mean	LoA
Within-therapist difference mod	2 (2%)	3 (3%)	7 (7%)	− 1.6 (2%)	− 8.0–4.9 (− 8–5%)
Within-therapist difference vig	2 (2%)	4 (3%)	6 (6%)	0.5 (1%)	− 11.5–12.5 (− 12–13%)
Between-therapist difference mod	5 (5%)	7 (7%)	31 (31%)	1.5 (2%)	− 18.7–21.8 (− 19–22%)
Between-therapist difference vig	2 (2%)	5 (5%)	15 (15%)	0.4 (0%)	− 12.5–11.8 (− 13–12%)

Robotic assistance (in %)	< 50%	< 75%	< 100%	Mean	LoA
Within-therapist difference mod	10 (5%)	10 (5%)	15 (8%)	− 0.4 (0%)	− 19.3–18.5 (− 10–10%)
Within-therapist difference vig	20 (10%)	28 (14%)	45 (22%)	− 3.3 (0%)	− 46.4–39.75 (− 23–20%)
Between-therapist difference mod	15 (8%)	25 (13%)	60 (30%)	8.0 (4%)	− 38.6–54.3 (− 19–27%)
Between-therapist difference vig	20 (10%)	35 (18%)	60 (30%)	8.0 (4%)	− 50.0–66.1 (− 25–33%)

The first three columns contain the cut-off values below which 50%, 75% or 100%, respectively, of the differences can be found. The cut-off values represent the median, the 75%-quartile and the maximal difference for both the moderate (mod.) and the vigorous (vig.) intensities. The other two columns represent the Bland–Altman statistics with the mean and the upper and lower limits of agreement (LoA). The values in the brackets contain the differences as percentage of the maximum difference possible

### Between-therapist agreement

For between-therapist agreement, differences in gait speed settings were similar to the within-therapist differences (Table 2). The differences in bodyweight support were slightly larger between therapists than within therapists (Table 2). Robotic assistance differed more than 25% between therapists in half of the participants but, in contrast to the within-therapist differences, this was the case in both the moderate and the vigorous condition.

The Bland–Altman statistics revealed a relatively even distribution with mean values close to 0. The exception were robotic assistance settings where the difference between therapists was slightly skewed. The distribution of the individual differences is presented in Fig. 2.

The parameter settings for the moderate and the vigorous condition differed significantly for all three parameters. Participants walked significantly faster with lower bodyweight support and lower robotic assistance during the vigorous condition (Table 3).

**Table 3:** Statistics of condition comparison

Parameter	Mean of differences (vigorous–moderate)	Df	T	p	95% Confidence interval
Gait speed (km/h)	0.23 (6%)	51	− 10.00	< 0.001	[0.18, 0.28]
Body weight support (% BW)	− 7.1 (− 7%)	51	9.31	< 0.001	[5.5, 8.6]
Robotic assistance (%)	− 43 (22%)	51	8.90	< 0.001	[33, 53]

The table shows the mean of the differences between moderate and vigorous settings within patients together with the results of the paired t-tests between moderate and vigorous conditions. p-values were corrected for multiple testing by a Bonferroni correction and 95% Confidence Intervals were calculated. Round brackets contain the mean of differences as percentage of the maximum possible difference

## Discussion

We found a high agreement for gait speed and bodyweight support settings as indicated by the small absolute differences. This means that therapists select very comparable gait speed and bodyweight support settings for the same patient in two consecutive trainings when given a similar training goal. This agreement was lower for robotic assistance. In general, the within-therapist agreement was slightly higher than the between-therapist agreement. These findings hold true for both therapy conditions, despite the statistically significant differences in parameter settings between the two conditions.

### Robotic assistance is more difficult to adjust than bodyweight support and gait speed

As within-therapist agreement was evaluated on two different days, it could be affected not only by variability in the therapist’s decision making but also by the day form of the patients. However, the agreement of gait speed and bodyweight support was very high. This suggests that the therapists estimated the physiologic abilities of the patients very similarly on both days. The agreement for robotic assistance was high in the moderate condition but lower in the vigorous condition suggesting that it was harder for therapists to estimate the impact of robotic assistance on the patient behavior. Changes in treadmill speed and bodyweight support usually lead to an immediate feedback: If bodyweight support is decreased, patients have to carry more load to prevent a collapse of the knee of the stance leg; if the treadmill speed is increased, patients have to speed up to prevent stumbling. Both changes are easily recognizable by the therapist (visually and/or acoustically). The robotic assistance setting does not have such a deterministic nature and patients can show diverse reactions to changes in this parameter. Technically, in the case of Guidance Force, for example, the applied torque is a function of the deviation from a target position. A reduction of Guidance Force merely adjusts the scaling of stiffness and damping, meaning that in both cases similar torques can occur [22]. In the case of a lower Guidance Force, the same torque just occurs at a larger deviation of a target trajectory, and only if the torque becomes too big or the deviation too large, a safety stop occurs. This means that a decrease in Guidance Force allows more kinematic freedom before a safety stop occurs, but it does not automatically imply less assistance. Thus, the effect of the robotic assistance is closely linked to the patient’s contribution and the instructions patients receive from the therapist. In the present study, participants were instructed to contribute actively to the movement, but instructions were tightly controlled. Thereby, we limited the therapist’s ability to verbally guide the patient, and variable reactions by the patient could have had an impact on the therapist’s choice of robotic assistance.

### The agreement on parameter settings is lower between therapists than within therapists

For the between-therapist agreement, similar observations can be made. The fact that therapists seemed to perceive similar combinations of gait speed and bodyweight support as suitable for the patients, despite the large variability between the chosen parameters for different patients, indicates that they estimated the individual capacity of the patients similarly. However, the choice of an appropriate robotic assistance was larger between therapists than within therapists even for the moderate condition.

If an increasing fatigue and decreasing motivation would be responsible for the discrepancy in robotic assistance, the second therapist would have to select a higher robotic. However, there was no systematic difference. While differences in the personal relationship between the different therapists and the patient could still affect patient behavior, it might again be the therapist’s understanding of robotic assistance that influences the parameter choice. The technical complexity of the closed-loop control requires at least some understanding of how the motor torques are influenced by the patient’s behavior. For therapists with little or no background in control engineering, this might be difficult to understand, especially because the device interface of the Lokomat does not inform the therapists on the deviations and/or actually applied torques. This complexity is further increased by the fact that the two different control modes, Path Control and Guidance Force, act in a superposed way [23]. In addition, therapists could employ different strategies and decide to focus more on strength training against the resistance of the Lokomat with a higher Guidance Force or active exploration with a lower Guidance Force and more gait variability. Both cases would be legitimate forms of challenging patients, but result in differences in robotic assistance.

### Comparison of intensity conditions

The significant difference between the moderate and vigorous condition (Table 3) in combination with the large variability of the chosen parameter settings between patients (Fig. 2) suggests that therapists use parameter settings not only to individualize the therapy for each patient, but also to modulate the intensity within a therapy session. The therapy parameter choice likely depends on whether therapists aim to sustain an effort for a prolonged period of time, for example to improve muscular endurance, or for short intervals to improve strength [24]. The two conditions investigated here likely mark only two, although distinct, examples and therapists might also use other combinations in a normal therapy session, considering different factors, like therapy goals, the patient motivation, or adherence to the therapy.

### Limitations

Some limitations have to be considered when interpreting the present study results. Firstly, there will always be a gap between a research setup that is highly standardized and the real-world therapy situations that allow for more flexibility. We strongly believe that in order to draw clinically useful conclusions, research has to mimic real-world therapy as closely as possible. We have tried to ensure this by specifying two conditions that may well occur in everyday practice. Irrespectively, at least some standardization of the task was necessary to achieve comparable conditions. However, the task was formulated in a way that gave a lot of flexibility to the therapists to take personal factors of the patients into account.

Secondly, no final conclusion can be drawn on the relative importance of the differences across parameters. However, van Kammen et al., showed in a series of studies that a change from 50% bodyweight support to 0% bodyweight support altered the muscle activation amplitudes to a similar extent as a change from 100% guidance force to 50% guidance force although a high bodyweight support seems to attenuate the effects of guidance force [13, 25, 26]. This suggests that ± 50% of robotic assistance (approximate limits of agreement) has indeed a bigger impact than ± 15% of bodyweight support. Moreover, the relatively low number of subjects can lead to a conservative estimate of the limits of agreement. This is especially relevant in cases where outliers are present. However, the observations are supported by the robust measures of the absolute differences. Furthermore, the results presented in this study are valid for the pediatric population and therapists that were trained based on the same philosophy at the Swiss Children’s Rehab. It might be that including different centers or different populations would lead to different results. Considering different centers would probably increase the variability of the therapists and it is more likely that the agreement would be weaker. Therefore, we firmly believe that many of the findings are also relevant for other centers, populations and even devices, if they are based on a similar tuning principle.

### Implications

While in the present study, only the Lokomat was investigated and its specific modality of robotic assistance, further strategies exist to apply robotic support therapy. These strategies have different advantages or disadvantages [27]. In addition to assistance, which is the main principle of the Lokomat, there are modalities, like resistance, adding noise or even augmenting errors from target trajectories. As more modern robotic devices that implement various control strategies and tuning parameters enter the market, the choice of an appropriate combination for a particular patient becomes even more important. Although some algorithms to automatically adjust the different parameters exist [28, 29], the current generation uses a single objective for parameter optimization, e.g. deviation from the desired trajectory in case of the Lokomat [30]. It is unlikely that such an approach can account for the variety of deficits that contribute to an impaired walking function and are differently pronounced in each patient. Compared to conventional therapy, this also alters the work of therapists from actively providing haptic interactions with the patients themselves to selecting therapy parameters and instructions appropriate for a patient-specific rehabilitation goal [10]. This is not straight forward as interactions between the parameters exist [13]. Consequently, changes in patient behavior when changing one parameter can be falsely attributed to the patient capacities whereas in fact they are caused by a unsuitable parameter combination that produces this behavior [28].Therefore, future research should aim to better support therapists at this task. This warrants that therapists are empowered to understand the range of behaviors patients can exhibit with relation to a mode of haptic training and how they can optimally employ other means, like for example, verbal instructions or virtual biofeedback to achieve physiological reactions in line with the participants’ therapy goal.

In a recent literature review, we were able to show that in most clinical studies, which investigated the effectiveness of robot-assisted gait therapy, therapy content received insufficient attention [8]. The present study further emphasizes the importance of the therapy content by showing that the preferred parameter settings are dependent on the intensity therapists want to achieve. These preferences are consistent for gait speed and bodyweight support, but not for robotic assistance even if therapists are similarly trained. However, robotic assistance is the only parameter that does not exist in traditional treadmill training. The large variability in the selected robotic assistance might also help to explain why a recent study found that bodyweight support and gait speed were more important in predicting outcomes than robotic assistance [31]. A more consistent selection of robotic assistance could also increase the relevance of this parameter for the prediction of clinical outcomes.

From a clinical perspective, it is important that enforcing a higher agreement of robotic assistance is not necessarily beneficial (e.g. by noting down parameter settings and strictly enforce a progression) as variable reactions would be still possible. Especially in the case of within-therapist agreement, some variability might actually be desired to account for day to day differences. Instead, therapists should aim to improve the between-therapist agreement (1) by linking the choice of robotic assistance to the individual therapy goals of the patients and (2) by closely guiding the patients with instructions during walking. Thereby, therapists might achieve an improved predictability of the patient behavior, which could potentially lead to an improved effectiveness of this therapy form.

## Conclusion

With this study, we were able to show that therapists show a high agreement their choice of parameters that have a very clear effect (changing walking speed, taking over more weight), but have more difficulties with robotic assistance, which has a more ambiguous effect. This might be attributed to the technical nature of robotic assistance, but also to the variable behavior of the patient (motivation or understanding of the task) or different intentions of the therapist (therapy goals or strategy). To harmonize the results of the personalization, individual therapy tasks and instructions should be taken more into account. Future work should focus on whether this can improve the predictability of how patients react to parameter changes and how robotic assistance can be best employed in combination with instructions to steer these reactions.

## Supplementary Information

**Additional file 1**. Standardised instruction.

**Additional file 2**. Patient characteristics.

**Additional file 3**. Study data.

## Data Availability

The datasets supporting the conclusions of this article are included in the supplemental material (see Additional file 3).
