# Supplementary material for: Within- and between-therapist agreement on personalized parameters for robot-assisted gait therapy: the challenge of adjusting robotic assistance
Source: J Neuroeng Rehabil. 2023 Jun 20;20:81. doi: 10.1186/s12984-023-01176-x (PMC10283166; doi:10.1186/s12984-023-01176-x)
Supplement: Supplementary file 1 — Additional file 1. Standardised instruction. [file 12984_2023_1176_MOESM1_ESM.docx]

Supplementary material 1: Standardized instructions

“Try to relax hands and arms.”

“Keep your trunk stable if possible.”

“Try to extend your leg.”

“Try to walk actively.”

“Continue as up to now! You are doing well.”

“You have 30s left.”
